# On Uterine Hæmorrhage and a New Method of Treatment

**Published:** 1885-02-20

**Authors:** Richard Richardson

**Affiliations:** Rhayader


### ON UTERINE HÆMORRHAGE AND A NEW METHOD OF TREATMENT.

Read in the Section of Obstetric Medicine, Belfast meeting, British Medical Association,

BY THE LATE RICHARD RICHARDSON, L.R.C.P., RHAYADER.

I shall not take up your time in considering the physiology and pathology of uterine hæmorrhage, but shall devote attention more particularly to the treatment which has been most successful in my hands during a period of twenty years. I was anxious to find a reliable remedy which could be easily applied without any apparatus; these advantages I found in iron-alum when applied in crystals of the size of a hazel-nut, or even larger in a severe case.

It is to be introduced with the finger up to the os uteri (and not into it), and there allowed to remain. The uterus will at once contract, a firm coagulum is formed, and the hæmorrhage at once ceases. Iron-alum is also antiseptic, as I have removed clots on the fourth and fifth day after its application which were quite free from any disagreeable odor. In a case of very severe hæmorrhage, two or three days afterward I inject a little warm water (to which may be added, if you like, a little Condy's fluid) and remove the clots. It is perfectly free from danger, and I have never known it to fail.

I have a record of 82 cases of uterine hæmorrhage where this remedy was applied without a single failure; menorrhagia, 10 cases; metrorrhagia, 18; abortions, 15; accidental hæmorrhage, 7; unavoidable hæmorrhage, 4; post partum hæmorrhage, 22; secondary hæmorrhage, 6.

In post partum hæmorrhage, it is advisable always to clear the uterus of clots or any portion of placenta before applying the crystal; also, in accidental hæmorrhage, when there is detachment of placenta, should the case appear to be one where there is no chance of carrying it to full term, the membranes ought to be punctured, and the iron alum applied in the usual way. There is no fear of any very great hæmorrhage coming on afterwards; in a slight case, the iron-alum will stop it; but, if the hæmorrhage should recur, it would be advisable to have recourse to puncturing the membrane, and induce labor. By way of comparison, I shall here enumerate most of the local remedies hitherto used in uterine hæmorrhage; namely, the tampon, compression. friction, galvanism or electricity, ice, injection of hot and cold water, cold water applied to the vulva, cold douche on the abdomen, pressure on the abdominal aorta, and last, but not least, the injection of liquor perchloridi. Most of these require an apparatus for their application, which may not always be at hand; and, in addition, some time would be taken in their preparation and administration. Furthermore, there is the always present danger of injecting a styptic into the open mouths of the uterine vessels; also, cold applications, when the body is already too cold, must be injurious. Now, iron-alum does not require any apparatus, or any preparation, as it is ready at hand; it will bring on immediate contraction of the uterus, which is the chief aim and object in the treatment of these cases; as remarked before, it does not require to be introduced into the uterus, only into the vagina, close to the os uteri, and there left.

The preparation is both cheap and effectual. I had some pessaries of it prepared by Messrs. Ferris & Co., which I found expensive, and with no better than the crude crystal; I therefore discontinued them.

I find the crystals made with ammonia more permanent than those made with potash, and therefore more to be preferred. Since I began using the crystals I never go to a case of labor without them; I have also used the pieces (too small to be introduced) tied up in a piece of muslin, leaving the ends of the string hanging outside, so as to more readily remove the alum on the following day. This method answers quite as well. One of my former assistants, Mr. G. Tombs, of Llanwrtyd Wells, wrote to me:

"I have very great faith in iron-alum since I learnt its value from you. I have used it very extensively, and can testify to its efficacy in uterine hæmorrhage. I have applied it in abortion, accidental, and post partum hæmorrhage, with the best result. In fact, in my experience I have not known it fail. I never go to a midwifery case without taking iron-alum with me."

Another old assistant, Mr. J. W. Hinings, of Bromyard, says:

"I have used the iron-alum in three cases of milder post partum hæmorrhage, too severe to be left alone, and have been satisfied with its operation."

My son, Mr. F. L. C. Richardson, says: "I use iron-alum, when there is the least tendency to uterine hæmorrhage in post partum cases, as a safeguard; but I found it invaluable in four extreme cases, as they were in a moribund state. I never undertake a midwifery case without bringing iron-alum along with me, as a reliable companion to ergot and tincture of opium."

My friend, Mr. Talfourd Jones, of Brecon, writes to me as follows: "Since you first recommended to me, some time ago, the local use of iron-alum in uterine hæmorrhage, I have used it on several occasions in cases of threatened abortion accompanied by troublesome bleeding, and I have found the remedy extremely useful and valuable, very certain and rapid in its action. It is well to bear in mind that it is apt to occasion considerable soreness of the vagina, and in one instance it gave rise to vaginitis."

The soreness after the application, referred to by Dr. Jones, I have never yet met with. It is probable, if a person wished to make an examination very soon after its application, there would be great inflammation of the parts, and in introducing the fingers some pain would undoubtedly be occasioned. It is better to wait until the following day to do so, having first of all sent up an injection of warm water to remove clots, etc.

I have also applied the remedy in four cases of uterine cancer, where the hæmorrhage was very considerable and frequent. It controlled the bleeding and was re-applied on its recurrence. It also acted as a good antiseptic in these cases, and was the means of making the patients' lives more comfortable during the last six months of their sufferings. I have used it in several cases of leucorrhœa with great benefit. I wish my medical brethren to give it an unbiassed trial; and some, who are engaged in this specialty in large towns, would have ample means of doing so, as they would be likely to meet with more cases in twelve months than I would in ten years, owing to the difference in population.

Finally, I would supplement the treatment of hæmorrhage by constitutional medicines, such as are generally recommended; namely, ergot, opium, digitalis, gallic acid, sulphuric acid, turpentine, acetate of lead and transfusion and hypodermic injection of sulphuric ether in collapsed cases. Of these, the most reliable remedies are the first two and the last two.—*British Medical Journal.*

